# Immune-neural crosstalk in chronic low back pain: from intervertebral disc degeneration to pain sensitization

**DOI:** 10.3389/fimmu.2026.1926648

**Published:** 2026-09-14

**Authors:** Jinjin Wei, Zhiyun Guan, Yaobin Long, Dongmei Huang, Yijin Zhao, Chongwu Xiao

**Affiliations:** 1 Department of Rehabilitation Medicine, The Second Affiliated Hospital of Guangxi Medical University, Guangxi Medical University, Nanning, China; 2 Department of Nursing, Beihai People’s Hospital, Beihai, China; 3 Center for Rehabilitation Medicine, Zhujiang Hospital, Southern Medical University, Guangzhou, China

**Keywords:** chronic low back pain, cytokines, dorsal root ganglion, immune-neural crosstalk, intervertebral disc degeneration, microglia, neuroinflammation, nociplastic pain

## Abstract

Chronic low back pain (CLBP) is a leading cause of disability, yet radiographic degeneration correlates poorly with symptoms. This mini-review examines how immune-neural crosstalk may link intervertebral disc degeneration to pain initiation and persistence. Degenerated nucleus pulposus and annulus fibrosus cells develop inflammatory and catabolic phenotypes and release mediators that may promote neural and vascular ingrowth, sensitize nociceptors, and increase dorsal root ganglion neuron excitability. Sustained peripheral input may engage spinal glial pathways and contribute to central sensitization. Pain may persist through expectation, learning, affective processes, and altered central prediction even when peripheral inflammatory input is limited. These interactions are bidirectional: immune signaling may affect mood, cognition, sleep, and threat processing, whereas stress, pain-related beliefs, and social context may modify immune and neural responses through autonomic and neuroendocrine pathways. Vertebral endplate inflammation, sometimes reflected by Modic type I changes, may mark an immune-associated phenotype. Although mechanistic and preclinical evidence supports targets such as NGF and pro-inflammatory cytokines, clinical benefit has been inconsistent, potentially because of safety concerns, patient heterogeneity, insufficient target engagement, and trial-design limitations. We propose a systems framework integrating inflammatory biomarkers, endplate imaging, quantitative sensory testing, neuroimmune imaging, and contextual measures. This framework may help distinguish inflammation-dominant, peripherally sensitized, central-sensitization-associated, nociplastic (pain arising from altered nociception), and mixed profiles. Validation requires prospective longitudinal studies, biomarker-stratified cohorts, and adequately powered randomized controlled trials.

## 1 Introduction

Chronic low back pain is one of the most disabling health problems globally. The 2021 Global Burden of Disease Study estimated that hundreds of millions of people worldwide are affected by low back pain; with an aging population, the disease burden is expected to continue to rise (1). Despite the large scale of the disease, current treatments are mostly nonspecific, relying mainly on analgesics, physical therapy, and interventional procedures, which do not address the biological mechanisms behind the pain in individual patients (2). Structural degeneration is also common on magnetic resonance imaging (MRI) in asymptomatic individuals. A systematic review by Brinjikji et al. showed that disc degeneration, decreased intervertebral disc height, and disc bulging were prevalent in asymptomatic individuals of all ages; in the elderly, the prevalence of some imaging features even exceeded 90% (3). Therefore, a degenerated intervertebral disc is not simply a worn-out mechanical structure. It can function as an active inflammatory microenvironment in which nucleus pulposus and annulus fibrosus cells produce cytokines, chemokines, prostaglandins, and matrix-degrading enzymes (4). This inconsistency constitutes the core paradox of disc-related pain: visible degeneration may provide a structural substrate for pain, but it is neither necessary nor sufficient to explain the chronic pain experience. Local immune events can initiate or amplify pain when they engage peripheral and central neural pathways; however, ongoing local inflammation and disc nociceptive input may not be the sole determinants of every pain episode once pain has become chronic. Experimental work shows that expectations and context can alter pain and pain-related brain activity and that non-nociceptive social threat can recruit regions that overlap with physical pain processing (5, 6). Disc-derived mediators can promote nerve ingrowth into tissues that were not originally innervated, sensitize nociceptors, and enhance the excitability of dorsal root ganglion (DRG) neurons, thereby strengthening peripheral nociceptive input (4). This immune-neural continuum is the main tissue thread of this article. Rather than listing every inflammatory mediator reported in intervertebral disc tissue, this review traces the pathway from disc inflammation through peripheral sensitization to central amplification, while also considering centrally maintained pain and the opportunities these interacting processes provide for mechanism-guided therapy.

## 2 Immune activation in degenerating intervertebral discs

Healthy intervertebral discs are sparsely vascularized and relatively isolated from systemic immune surveillance. This protective state is gradually destroyed as degeneration progresses. Mechanical overload, endplate injury, annulus fibrosus tearing, nutritional deficiencies, oxidative stress, and aging can all contribute to the shift of intervertebral disc cells towards an inflammatory and catabolic state (4, 7). Risbud and Shapiro’s landmark review linked disc-derived cytokines to matrix destruction and pain signals, establishing the concept that degenerated discs are immune-active organs rather than passively undergoing structural failure (4).

Subsequently, the relevant molecular evidence has become more detailed. Shamji et al. reported IL-17 immunoreactivity in more than 70% of surgical tissue fields, whereas IL-4, IL-6, IL-12, and IFN-γ immunoreactivity was modest but higher in herniated than in degenerated intervertebral disc specimens (7). Wang et al. reviewed the roles of IL-1β and TNF-α in intervertebral disc degeneration, elucidating how these cytokines induce the expression of matrix metalloproteinases (MMPs) and a disintegrin and metalloproteinase with thrombospondin motifs (ADAMTS) enzymes, inhibit matrix anabolic metabolism, and amplify inflammatory signals through NF-κB (8). De Luca et al. found that after IL-1β priming, intervertebral disc and endplate cells exhibited a specific inflammatory response profile, and potential therapeutic targets were identified through transcriptomic analysis (9). Tu et al. used single-cell transcriptomics to depict the multicellular ecosystem of the nucleus pulposus during the degenerative process, revealing cellular state transitions and inflammatory subsets that could not be identified by whole-tissue analysis (10).

The NF-κB signaling pathway is particularly noteworthy as a hub. In an inducible mouse model, prolonged NF-κB activation in intervertebral disc cells promoted macrophage recruitment and severe degeneration in caudal discs, whereas lumbar discs did not develop the same degenerative phenotype (11). These findings provide *in vivo* evidence that sustained NF-κB activation can drive inflammatory and structural changes, while also highlighting important regional and model-specific limitations. A separate study found that chemerin-TLR4/CMKLR1-NF-κB signaling is another pathway connecting intervertebral disc stress with innate immune activation (12).

Inflammasome biology further adds to the complexity of this mechanism. Chen et al. found that the levels of NLRP3, caspase-1, and IL-1β were elevated in degenerated human intervertebral discs, and their expression was correlated with the degeneration grade (13). He et al. showed in cell and animal models that Cutibacterium acnes (formerly Propionibacterium acnes) can induce nucleus pulposus cell pyroptosis through an NLRP3-dependent pathway (14). These findings support a possible inflammatory mechanism but do not establish the prevalence, causal importance, or clinical relevance of occult disc infection. Their mechanistic significance lies in NLRP3-dependent pyroptosis with increased IL-1β and IL-18 signaling.

Immune cell infiltration adds another layer. Macrophages can enter herniated or damaged intervertebral disc tissue and interact with resident cells. IL-17 produced by T cells and innate-like lymphocytes can amplify the inflammatory cascade and matrix degradation in the intervertebral disc microenvironment (15). Takada et al. found in an experimental model of intervertebral disc herniation that the interaction between the intervertebral disc and macrophages can induce mechanical hyperalgesia and cytokine production, directly linking immune infiltration with pain behavior (16). The key point is that intervertebral disc inflammation is not caused by a single cytokine, but by a network reaction involving intervertebral disc resident cells, infiltrating immune cells, danger signal sensing pathways, and matrix-derived feedback (**Figure 1**).

**Figure 1:**
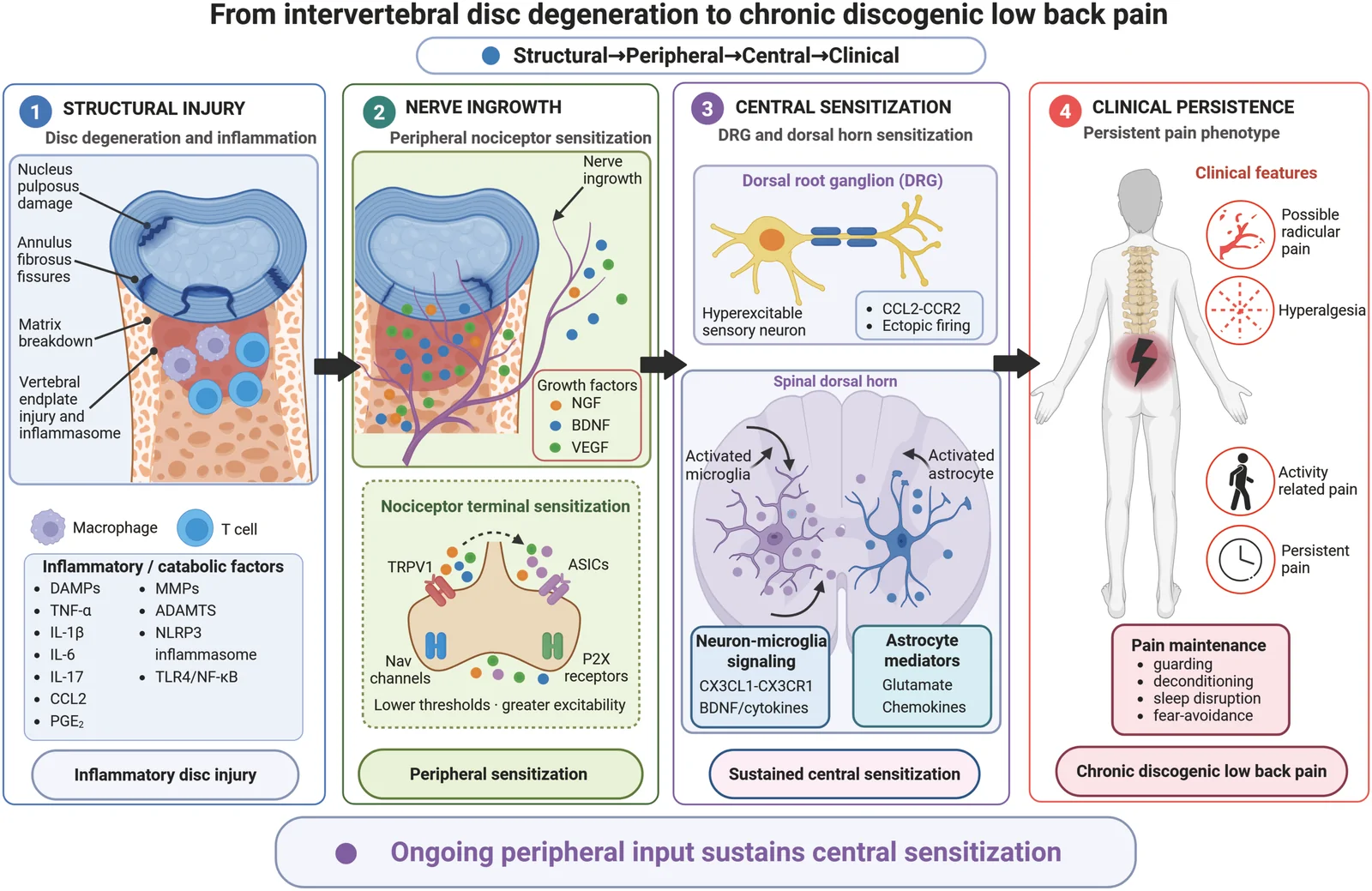
Immune-neural mechanisms linking intervertebral disc degeneration to chronic low back pain. DAMPs, damage-associated molecular patterns.

## 3 From disc inflammation to peripheral pain sensitization

In normal adults, intervertebral discs have limited innervation, mainly confined to the outer layer of the annulus fibrosus. Painful degeneration, however, is associated with nerve ingrowth into areas that are normally uninnervated. A classic study by Freemont et al. published in The Lancet found that in painful degenerated discs, sensory nerves can ingrow into the inner third of the annulus fibrosus and even into the nucleus pulposus, accompanied by vascular ingrowth (17). This finding supports an association between deep nerve ingrowth and painful disc degeneration and provides a structural basis for direct nociceptive signal transduction within the disc.

Nerve ingrowth is not spontaneous. Degenerated disc cells produce neurotrophic factors, especially NGF and BDNF, as well as angiogenic mediators such as VEGF, which may promote neural and vascular ingrowth into damaged tissue (18). Richardson et al. found through co-culture experiments that degenerated human nucleus pulposus cells can promote neurite outgrowth in neural cells, supporting the idea that the disc itself can actively recruit innervation (19). Inflammatory mediators then alter the response of these nerve fibers. TNF-α, IL-1β, IL-6, PGE2, NGF, and chemokines can lower the activation threshold of nociceptors and enhance their response to mechanical and chemical stimuli. Lai et al. demonstrated that annular puncture with intradiscal TNF-α enhanced pain-related behavior in a model of intervertebral disc degeneration (20). In an ISSLS Prize-winning study, Miyagi et al. observed age-dependent increases in disc innervation and sensory nervous system plasticity in SPARC-null mice that developed behavioral signs of low back pain (21). The DRG is a key relay in this transition. Disc-derived inflammatory mediators and the acidic degenerative microenvironment can directly sensitize primary sensory neurons. Stover et al. found that degenerative intervertebral disc-conditioned media and acidic pH sensitized sensory neurons to cyclic tensile strain, indicating that the degenerative disc microenvironment can directly alter neuronal mechanosensitivity (22). CCL2-CCR2 signaling within the DRG is involved in maintaining nociceptive sensation: Zhu et al. demonstrated that this chemokine axis can maintain neuropathic pain in a lumbar disc herniation model (23). Xie et al. further showed that spinal CCL2 can promote central sensitization and long-term potentiation through a CCR2-dependent synaptic plasticity mechanism (24). Sessler et al. found that CX3CL1-CX3CR1 signaling, as a neuron-microglia communication pathway, is crucial for neuronal sensitization in an experimental nonspecific myofascial low back pain model, thus establishing a link between peripheral immunochemical changes and spinal cord pain processing (25).

## 4 Neuroimmune amplification and central sensitization

Continuous peripheral nociceptive input can remodel spinal cord pain processing. In preclinical pain models, microglia, the resident innate immune cells of the central nervous system, can contribute to pain amplification, and microglia-derived BDNF provides one pathway through which immune activation may alter neuronal inhibition (26, 27). In a rodent peripheral nerve-injury model, Coull et al. showed that microglial BDNF downregulated the neuronal potassium-chloride cotransporter KCC2 and shifted the anion gradient, thereby weakening GABAergic inhibition and promoting neuropathic pain (26). Although biologically informative, this mechanism has not been established as a causal pathway in human chronic low back pain. More broadly, activated microglia and astrocytes in the dorsal horn release mediators such as IL-1β, TNF-α, IL-6, CCL2, prostaglandins, and BDNF, enhancing synaptic transmission, altering inhibitory tone, and promoting central sensitization (28, 29).

Astrocytes may be particularly important for pain maintenance. They can continuously drive cytokine and chemokine signaling, regulate extracellular glutamate, and support long-term alterations in dorsal horn excitability (29, 30). Human neuroimaging studies have begun to provide translational evidence. Using [11C]PBR28 positron emission tomography (PET), in which the radioligand binds to the translocator protein (TSPO), Loggia et al. found increased thalamic signal in patients with chronic low back pain compared with controls (31). Because TSPO is not cell-type specific, this finding is consistent with, but does not uniquely establish, glial activation in the living human brain. Aoyagi et al. identified a subgroup of patients with chronic low back pain who showed clinical and quantitative sensory features consistent with central sensitization (32). Arendt-Nielsen et al. reviewed how manifestations such as increased temporal summation and impaired conditioned pain modulation can be assessed across chronic pain conditions (33). Zhang et al. reported that pharmacological glial inhibition prevented and reversed latent sensitization of dorsal horn neurons in a rat model of low back pain (34). Because these pharmacological agents are not fully cell specific, the findings support a contributory role for spinal glial signaling but do not establish that glia are necessary or sufficient for pain chronification. These findings motivate investigation of centrally targeted therapies in patients with clinical features of central sensitization; however, current sensory and imaging measures do not establish a validated neuroimmune treatment phenotype. Peripheral and central immune mechanisms should not be viewed as separate compartments. They form a feedback loop in which disc inflammation, DRG sensitization, spinal glial activation, and descending regulation interact. Patients whose pain is primarily driven by local disc inflammation may be fundamentally different from those whose pain is maintained by central sensitization despite minimal sustained peripheral input. Determining where each patient falls within this continuum is a challenge for treatment selection, and a single MRI scan is insufficient.

Importantly, ongoing local immune activity or disc nociceptive input may not be the sole determinant of every chronic pain episode. Experimental studies show that expectations can alter pain and pain-related brain activity (5) and that social rejection can recruit some regions also engaged during physical pain processing (6). Although these experiments do not directly establish the mechanisms that maintain chronic low back pain, they support the possibility that threat appraisal, attention, learning, and prediction can amplify pain when peripheral tissue activity is reduced. Such pain is neither imaginary nor evidence of malingering; it is a real experience generated by distributed neural systems. In one randomized trial of carefully selected patients with primary chronic back pain, pain reprocessing therapy (PRT), a cognitive, somatic, and exposure-based intervention, produced larger and more durable improvements than open-label placebo or usual care (35). Because treatment response does not establish the absence of peripheral nociceptive input or identify a neuroimmune mechanism, this result supports psychologically informed treatment in selected patients but should not be interpreted as proof of a purely centrally maintained pain state.

Psychosocial processes are therefore not an external overlay on neuroimmune biology but part of a bidirectional feedback system. In the immune-to-brain direction, peripheral or central cytokine signaling can alter fatigue, sleep, mood, social connectedness, attention, and threat processing. In a randomized human endotoxin experiment, increases in IL-6 and TNF-α were accompanied by greater depressed mood and social disconnection (36). In the brain-to-immune direction, perceived threat, anxiety, pain catastrophizing, social stress, and disrupted sleep may modify autonomic and hypothalamic-pituitary-adrenal signaling, immune-cell activity, and cytokine reactivity. In a small chronic-pain study, pain catastrophizing was associated with amplified IL-6 responses to painful stress, although the clinical significance remains uncertain (37). These loops may reinforce hypervigilance, avoidance, and central sensitization, but they are variable rather than universal. Longitudinal studies with repeated psychosocial, immune, and sensory measures are needed to establish temporal direction and identify modifiable mediators (29, 30).

## 5 Vertebral endplate inflammation, Modic changes, and pain phenotypes

Painful degeneration can also originate from the vertebral endplates and adjacent bone marrow. Modic changes, especially type I changes characterized by edema-like marrow signal on MRI, are associated with inflammation-dominant biological processes and may identify a subgroup in which immune-mediated mechanisms warrant further study (38). Ohtori et al. observed TNF-immunoreactive cells and PGP 9.5-positive nerve fibers in the vertebral endplates of patients with discogenic low back pain, providing histological evidence for the simultaneous presence of inflammation and innervation in the endplates under painful degeneration (39). The ISSLS Prize-winning study by Dudli et al. identified pro-inflammatory and fibrogenic crosstalk between Modic bone marrow and adjacent intervertebral discs (40). Molecular studies further support the involvement of immune mechanisms in this phenotype. Vigeland et al. linked short tau inversion recovery (STIR) signals on MRI in patients with chronic low back pain and Modic changes with gene expression patterns suggesting immune involvement (41). In a case-control comparison, Gjefsen et al. found that serum macrophage migration inhibitory factor (MIF) contributed to the separation of patients with chronic low back pain and Modic changes from healthy controls; because patients with chronic low back pain without Modic changes were not included, its ability to identify a Modic-specific subgroup remains untested (42). Chen et al. identified a COX-2/PGE2/EP-4 signaling axis associated with patient-reported pain symptoms in vertebral endplate defects, thus linking a specific prostaglandin pathway to a clinically identifiable structural abnormality (43).

These results should still be interpreted with caution. Modic changes are heterogeneous, and not all radiographic findings are clinically painful. A systematic review and meta-analysis by Lambrechts et al. found that Modic changes were associated with statistically worse back pain and disability scores, but the mean differences were below established thresholds for clinical importance (44). The value of the Modic framework lies not in identifying all pain patients, but in identifying a subgroup where endplate-mediated immune activation may form a unique pain phenotype. This subgroup may become a more tractable population for anti-inflammatory treatment or biomarker-guided clinical trials.

## 6 Pain phenotyping: integrating existing models with a systems neuroimmune framework

Pain phenotyping in CLBP is not a new endeavor. A systematic review of 43 studies found that phenotype definitions have used pain location, severity, quality, and duration; pain impact including disability, sleep, and fatigue; psychological, behavioral, and social factors; and sensory profiles such as pain sensitivity and sensitization. However, the selected domains and methods varied substantially, and the review called for a standardized, comprehensive, and clinically feasible assessment framework (45). The Pain and Disability Drivers Management model provides one pragmatic biopsychosocial structure; in a retrospective rehabilitation cohort, its cognitive-emotional domain predicted change in disability, whereas nociceptive, nervous-system dysfunction, and cognitive-emotional domains were associated with baseline disability, but overall prognostic performance was limited (46). More recently, operational phenotyping has emphasized that a phenotype becomes clinically useful only when feasible inputs are linked to explicit decision logic, routing or escalation pathways, and measurable outcomes (47).

The immune-neural framework proposed here should therefore be added as a mechanistic layer to, rather than a replacement for, these established domains. A feasible core assessment could include pain distribution and impact, neurological examination, psychosocial and contextual measures, and structural and endplate imaging (38, 44, 45). Hypothesis-triggered add-ons could then test the suspected dominant driver: Modic or endplate imaging together with candidate systemic immune correlates associated with the Modic/endplate phenotype (38, 41, 42); radicular distribution and neuropathic signs for suspected peripheral neural involvement; and temporal summation, conditioned pain modulation, widespread sensitivity, and, in research settings, functional or neuroimmune imaging for central amplification (31–33). Because these processes interact, patients should be assigned probabilistically to inflammation-dominant, peripherally sensitized, central-sensitization-associated, nociplastic, or mixed profiles rather than forced into mutually exclusive bins. Nociplastic pain is defined as pain arising from altered nociception without clear evidence that actual or threatened tissue damage activating peripheral nociceptors or a lesion of the somatosensory system explains the pain (48). It is not synonymous with central sensitization and may coexist with nociceptive or neuropathic mechanisms.

To guide treatment, each phenotype must be tied to a testable action and reassessed longitudinally. A peripherally sensitized profile may justify research testing of NGF-targeted strategies, but it does not currently support routine prescribing. Tanezumab showed modest efficacy with important joint-safety limitations in a phase 3 CLBP trial (49); it has not been approved for CLBP. Separately, the European Medicines Agency refused its marketing authorization application for osteoarthritis pain because the benefits were not considered to outweigh the joint-related risks (50). In patients with radicular pain due to lumbar disc herniation, small trials of anti-TNF approaches have reported modest and methodologically heterogeneous signals that should not be generalized to nonspecific chronic low back pain (51, 52). A central-sensitization-associated or nociplastic profile may support graded activity and psychologically informed treatments such as PRT (35, 53). For mixed phenotypes, we propose sequenced multimodal care with repeated reassessment. These links are mechanistic treatment hypotheses, not validated prescribing rules. Prospective studies should test whether a composite systems phenotype predicts differential treatment response beyond conventional clinical risk stratification.

## 7 Therapeutic implications and translational barriers

Mechanistic studies nominate cytokine, NGF-TrkA, inflammasome, prostaglandin, and glial pathways as potential therapeutic targets (4, 13, 28). However, the supporting evidence is uneven. Much of it comes from reductionist cell systems or short-duration animal models that isolate one pathway but do not reproduce the age distribution, multimorbidity, treatment history, psychosocial context, and mixed pain mechanisms typical of chronic low back pain (30). Human tissue studies are commonly cross-sectional and cannot determine whether an inflammatory signal causes pain, reflects tissue damage, or marks a transient disease phase. Differences in tissue sampling, assay platforms, imaging definitions, and clinical outcomes further limit comparison across studies. Mechanistic plausibility should therefore be treated as target nomination, not as evidence that systemic or local inhibition will provide a favorable clinical benefit-risk balance.

The phase 3 tanezumab trial (NCT02528253) illustrates the gap between target validation and clinical utility. At week 16, 46.3% of patients receiving 10 mg tanezumab achieved at least 50% pain reduction, compared with 37.4% receiving placebo; the 5 mg dose did not meet the primary endpoint. During joint-safety follow-up through week 80, prespecified joint-safety events occurred in 2.6% of the 10 mg group versus 0% of the placebo group, and seven participants receiving 10 mg underwent total joint replacement (1.4%) (49). Several factors may explain the modest average benefit despite strong NGF biology. NGF is a shared sensitization pathway, but its involvement does not establish that it is the dominant pain driver in every enrolled patient. Systemic blockade also exposes joints unrelated to the index pain condition, and reduced protective nociception may contribute to a dose-related joint-safety trade-off. A substantial placebo response and enrollment without evidence of NGF-pathway activity may also have reduced the between-group contrast. Thus, a statistically positive trial can still fail the practical test of a clinically meaningful and acceptable benefit-risk profile in an unselected population.

Pro-inflammatory cytokine targets face a different but related set of barriers. TNF-α, IL-1β, IL-6, chemokines, and inflammasome products operate in redundant and time-dependent networks, so blocking one mediator may be bypassed by parallel pathways. Cytokine blockade may also be applied too late, after peripheral input has already produced durable neural plasticity, or to the wrong clinical compartment: acute, imaging-confirmed radicular pain is biologically different from heterogeneous nonspecific chronic low back pain. Drug access to the relatively avascular disc, uncertainty about local versus systemic delivery, and safety concerns associated with sustained immune modulation further narrow the therapeutic window. The anti-TNF trials cited here were small, used different agents and routes, and lacked biomarker confirmation of pathway activity (51, 52). These limited and non-comparable findings do not establish a reliable benefit that can be generalized to heterogeneous chronic low back pain; nor do they by themselves invalidate cytokine biology. Rather, they expose weaknesses in target timing, patient selection, delivery, statistical power, and trial comparability.

Exercise therapy has an established evidence base for chronic low back pain, although average improvements in pain and function are generally modest (53). Small and heterogeneous studies have reported changes in systemic inflammatory biomarkers after exercise or other nonpharmacologic interventions, but it remains unclear whether these changes mediate clinical benefit (54). In addition, a small cross-sectional exercise study linked salivary IL-6 reactivity to activity-related pain rather than demonstrating an anti-inflammatory treatment mechanism (55). The phenotype framework outlined above suggests that treatment selection should begin with the suspected dominant mechanism and the confidence in that assignment, while recognizing mixed states and repeating assessment over time. Imaging alone is insufficient, and neuroimmune biomarkers remain investigational rather than stand-alone prescribing tests. Clinical trials should therefore predefine composite phenotypes and test treatment-by-phenotype interactions rather than merely report overall mean effects (45–47) (**Table 1**).

**Table 1:** Candidate immune-neural pathways and proposed therapeutic implications in chronic low back pain.

Pathway	Main source	Pain relevance	Investigational implication
TNF-α/IL-1β (4, 8)	Disc cells; macrophages	Disc inflammation, matrix breakdown, nociceptor sensitization	Anti-cytokine or local anti-inflammatory strategies
CCL2-CCR2/CX3CL1-CX3CR1 (23–25)	DRG neurons and spinal astrocytes (CCL2); primary afferents and dorsal horn neurons (CX3CL1)	Experimental pain maintenance, spinal synaptic plasticity, and neuron-glia signaling	Chemokine-pathway modulation
NGF-TrkA (18, 19)	Disc and immune/stromal cells (NGF); sensory neurons (TrkA)	Nerve ingrowth and peripheral sensitization	Anti-NGF therapy or local NGF control
NLRP3/TLR4-NF-κB (12–14)	Nucleus pulposus and other intervertebral disc cells	Inflammatory signaling, matrix catabolism, and NLRP3-dependent pyroptosis in experimental disc-degeneration models	Inflammasome or innate-immune pathway targeting
PGE2/COX-2 (43)	Cartilage endplate tissue and cells	Association of COX-2/PGE2/EP4 expression with endplate defects and patient-reported symptoms	Nonsteroidal anti-inflammatory drugs (NSAIDs) or COX-2/PGE2 pathway modulation
Microglial BDNF (26, 28, 29)	Spinal microglia	Dorsal horn disinhibition and central sensitization	Microglia-BDNF signaling modulation
MMPs/ADAMTS (4, 8)	Intervertebral disc cells under cytokine stimulation	Cytokine-associated extracellular-matrix catabolism	Matrix-protective or anti-catabolic approaches
Bidirectional psychosocial-neuroimmune signaling (29, 30, 36, 37)	Cortical-limbic, autonomic/HPA, and immune systems	Affect, threat appraisal, cytokine reactivity, and pain persistence	Integrated psychologically informed, biological, and neural care

The pathway-treatment links shown are mechanistic hypotheses rather than validated biomarkers or prescribing rules; their predictive value requires prospective clinical testing.

DRG, dorsal root ganglion; NGF, nerve growth factor; BDNF, brain-derived neurotrophic factor; HPA, hypothalamic-pituitary-adrenal; PRT, pain reprocessing therapy.

Translation also depends on study design. Early-phase trials should demonstrate target engagement in the relevant tissue or neuroimmune compartment and establish dose, route, and safety before large efficacy studies. Later trials should use prospective phenotype enrichment or stratified randomization, predefine treatment-by-phenotype interactions, standardize clinically meaningful pain and function outcomes, control important co-interventions, and include follow-up long enough to assess durability and delayed harms. Biomarkers should be evaluated as reproducible multivariable classifiers rather than single-threshold prescribing tests, with external validation and adjustment for age, adiposity, medication use, comorbidity, sleep, and affective state. Adequate power is especially important because enrichment reduces the eligible population and interaction tests require larger samples than tests of an overall mean effect.

## 8 Conclusion

Chronic low back pain is not merely a structural consequence of intervertebral disc degeneration. It reflects an immune-neural continuum connecting disc inflammation, endplate immune activation, nociceptor sensitization, increased DRG excitability, activation of spinal microglia and astrocytes, and altered descending regulation. In some patients, learned expectations and threat-related processes may further sustain or amplify pain (5, 6, 35). Heterogeneity across this continuum helps explain why radiographic degeneration does not strictly correlate with pain intensity, why pain can persist or recur after the initial structural or inflammatory event has stabilized, and why therapies targeting a single site have limited efficacy in unselected populations.

Pain phenotyping has a substantial literature (45–47), but its definitions, measurement strategies, and ability to predict treatment response remain insufficiently standardized. The next step is therefore to make existing phenotype concepts more mechanistically interpretable, operational, and prospectively testable rather than to propose another unvalidated classification. The systems neuroimmune layer proposed here connects tissue and endplate inflammation, peripheral and central sensitization, and learning and contextual processes to established biopsychosocial domains and explicit treatment pathways. Composite profiles that integrate feasible clinical measures with hypothesis-triggered biomarkers and imaging may help distinguish inflammation-dominant, peripherally sensitized, central-sensitization-associated, nociplastic, and mixed profiles. Treatment matching should remain probabilistic and iterative, and proposed phenotype-treatment links require prospective validation before routine use. Without such validation and stratification, even mechanistically well-founded treatments may produce disappointing results in heterogeneous clinical trial populations.

A concrete validation agenda should include prospective longitudinal cohorts with repeated endplate and disc imaging, inflammatory biomarkers, quantitative sensory testing, and psychosocial assessments to establish temporal sequences rather than cross-sectional associations. Candidate classifiers should then be tested in biomarker-stratified or phenotype-enriched cohorts with prespecified thresholds and external validation. Adequately powered, multisite randomized controlled trials should randomize within strata, prespecify treatment-by-phenotype interactions, use standardized clinically meaningful pain and function endpoints, and report adverse events and durability. Embedded mechanistic substudies should test target engagement and mediation. Retaining biological samples and reporting results by timing, route, and phenotype would help distinguish an invalid target from a valid target tested in the wrong population, at the wrong disease stage, or with the wrong delivery strategy.
